# Silymarin-Enriched Biostimulant Foliar Application Minimizes the Toxicity of Cadmium in Maize by Suppressing Oxidative Stress and Elevating Antioxidant Gene Expression

**DOI:** 10.3390/biom11030465

**Published:** 2021-03-21

**Authors:** Hesham F. Alharby, Hassan S. Al-Zahrani, Khalid R. Hakeem, Hameed Alsamadany, El-Sayed M. Desoky, Mostafa M. Rady

**Affiliations:** 1 Department of Biological Sciences, Faculty of Science, King Abdulaziz University, 21589 Jeddah, Saudi Arabia; halharby@kau.edu.sa (H.F.A.); hsalzahrani@kau.edu.sa (H.S.A.-Z.); kur.hakeem@gmail.com (K.R.H.); halsamadani@kau.edu.sa (H.A.); 2 Botany Department, Faculty of Agriculture, Zagazig University, Zagazig 44519, Egypt; Sayed1981@zu.edu.eg; 3 Botany Department, Faculty of Agriculture, Fayoum University, Fayoum 63514, Egypt

**Keywords:** maize crop, performance, cadmium stress, antioxidant system, gene expression, plant extract, silymarin

## Abstract

For maize, the potential preventive role of foliar spraying with an extract derived from maize grain (MEg, 2%), silymarin (Sm, 0.5 mM), or silymarin-enriched MEg (MEg-Sm) in attenuating the stress effects of cadmium (Cd, 0.5 mM) was examined using a completely randomized design layout. Under normal conditions, foliar spraying with MEg, Sm, or MEg-Sm was beneficial (with MEg-Sm preferred) for maize plants, whereas the benefit was more pronounced under Cd stress. The use of Cd through irrigation water decreased plant growth traits, photosynthetic efficiency, including instantaneous carboxylation efficiency, Fv/Fm, and pigment contents, and hormonal contents (e.g., auxin, gibberellins, cytokinins including trans-zeatin, and salicylic acid). These undesired findings were due to an increase in Cd content, leading to increased levels of oxidative stress (O_2_^•^^−^ and H_2_O_2_), ionic leakage, and lipid peroxidation. Therefore, this damage resulted in an increase in the activities of nonenzymatic antioxidants, Sm, antioxidative enzymes, and enzyme gene expression. However, under Cd stress, although foliar spray with MEg or Sm had better findings than control, MEg-Sm had better findings than MEg or Sm. Application of MEg-Sm greatly increased photosynthesis efficiency, restored hormonal homeostasis, and further increased the activities of various antioxidants, Sm, antioxidative enzymes, and enzyme gene expression. These desired findings were due to the suppression of the Cd content, and thus the levels of O_2_^•^^−^, H_2_O_2_, ionic leakage, and lipid peroxidation, which were positively reflected in the growth and accumulation of dry matter in maize plants. The data obtained in this study recommend applying silymarin-enriched maize grain extract (MEg-Sm at 0.24 g Sm L^−1^ of MEg) as a spray solution to maize plants when exposed to excess Cd in soil or irrigation water.

## 1. Introduction

To maintain food security and crop productivity as well as to maintain sustainable agriculture, the accumulation of heavy metals on agricultural lands must be restricted. Crop plants must be qualified to cope with the adverse effects of heavy metals using novel strategies to minimize plant uptake or increase the plant’s resistance to their massive damage. Farmland contaminated with heavy metals is causing a dangerous decline in the efficiency of processes in plants concerning growth and productivity [1,2,3,4,5,6,7,8]. The increase in heavy metals in the soil stimulates oxidative stress linked to the overproduction of ROSs (reactive species of oxygen radicals). ROSs threaten plants by endangering various pathways related to both physiobiochemistry and molecular biology [8,9,10]. The decrease in plant growth due to heavy metals depends on some factors, including plant species, heavy metal concentration, growth conditions, and experimental conditions [11,12]. Among various heavy metals, the highest toxic effect of cadmium (Cd)-related pollution has been observed in wheat [1,3,6,10], pea [13], and rice [8,14].

Agricultural land and the plants cultivated on it have become severely threatened due to Cd toxicity worldwide [4,15]. Even with low concentrations, Cd is harmful to crop plants, and thus harmful to humans and animals that feed on these Cd-contaminated plants. After being absorbed by the root system of the plant, Cd easily transports into the shoot and adversely influences plant morphology and physiobiochemistry during all stages of the plant life cycle (e.g., germination, vegetative growth, and fruiting stages) [16]. The usual symptoms common to plants are stunted root and shoot, chlorosis of leaves, and a sharp decrease in biomass accumulation, all of which ultimately lead to plant death [9,10,11,12,13,14,15,16]. Cd accumulation greatly affects the absorption and transportation of almost all key nutrients in different parts of the plant [17,18,19]. These adverse events, particularly concerning the interference between the Cd metal and essential nutrients, can be attributed to the channel competition for nutrient uptake occurring at the molecular level [18].

Often, the endogenous antioxidant defense system is not sufficient for the plant to defend against environmental foes, including Cd. Thus, a plant extract known to be a biostimulant, such as maize grain extract (MEg), can be used as a foliar spray and/or seed priming solution to support plants to increase their tolerance to environmental opponents [3,20,21,22,23], including Cd stress [3].

Presently, MEg has been used to enhance plant efficiency under different stress conditions, including Cd stress, as it is an essential organic biostimulator rich in many growth-promoting substances for different stressed plants, such as antioxidants, phytohormones, and essential nutrients [3]. After applying MEg, plant morphology, physiology, and biochemistry have been positively modified along with stimulation of plant tolerance against damage of some stresses [3,20,21,22,23]. Therefore, MEg is a potent novel biostimulator to give stressed plants the power to resist damage from environmental opponents.

Among the most essential crops around the world, maize (*Zea mays* L.) is ranked third after wheat and rice. In developing countries, *Zea mays* is one of the key dietary food components because of its high nutritional value [24]. Due to the increasing environmental opponents caused by climate change, and the rapid and turbulent growth of industries and demographics, the yield of *Zea mays* is decreasing worldwide, and the issue has been exacerbated by the arrival of Cd in humans and animals [25], thus toxicity from Cd in *Zea mays* is a major concern.

As found in the preliminary study of the current investigation (Table S1), *Zea mays* (as a C_4_ crop) is more sensitive to Cd and more responsive to MEg than other species such as wheat [3], so it was selected for the present study. There are no investigations on the influences of MEg and Cd on *Zea mays*; however, only one paper has been published dealing with the influences of MEg and Cd, but it focused on *Triticum aestivum* [3]. To date, no investigations have been conducted with silymarin (Sm)-enriched MEg (MEg+Sm) for *Zea mays* grown under Cd stress. Therefore, this is the first investigation in which MEg+Sm was applied to leaves to encourage the growth of *Zea mays* under Cd stress. MEg lacks silymarin, so MEg was enriched with silymarin for this study.

This investigation was, therefore, aimed at studying the influences of MEg+Sm on plant growth, physiobiochemistry, enzyme activities, and enzyme-related gene expressions in Cd-stressed *Zea mays*. To fulfill this hypothesis, a set of morphophysiobiochemical and molecular indices was identified to investigate MEg+Sm-induced stress tolerance to Cd in *Zea mays*.

## 2. Materials and Methods

### 2.1. Plant Material, Experimental Description, and Layout

Maize seeds (cv. Hybrid 306) were secured from the ARC (Agricultural Research Center), Egypt. A 1% solution of sodium oxychloride (NaOCl, 5%) was used to sterilize the seed surface for 2 min, and distilled water was then utilized to thoroughly wash the seeds. Using sterilized Petri dishes (12 cm in diameters), the sterilized seeds were germinated using moistened filter papers at 20 °C under 16 h/8 h light/darkness for 7 days. Five germinated seedlings were carefully transplanted in each pot filled with 10 kg of ion-free sand moistened with a nutrient solution. As detailed in [26], the composition of the nutrient solution used for watering *Zea mays* is presented in Table 1. Every other day, watering was applied to the seedlings with this nutrient solution.

Thinning into three seedlings of similar size per pot was implemented after four irrigation times. The following controlled conditions were applied for seedling growth: Photon flux of 390 mE m^−2^ s^−1^ at plant height with dark/light regime 14/10 h at 20/24 °C was applied under a relative humidity of 65–70%.

Cadmium (Cd; Sigma-Aldrich, St. Louis, MO, USA) treatment was started two weeks after transplantation. The cadmium was applied to plants at a concentration of 0.5 mM using cadmium sulfate (CdSO_4_) with the nutrient solution added to the seedlings every other day. The 0.5 mM Cd was selected for the main study based on our initial study. It was found that 15 irrigation times applied throughout the experiment with 0.5 mM Cd had the most damage to the maize plants, whereas 15 irrigation times with a concentration of more than 0.5 mM killed the plants (Table S1). Foliar spraying with maize grain extract (MEg), silymarin (Sm; Sigma-Aldrich, St. Louis, MO, USA), or silymarin-enriched MEg was implemented on the seedlings one week after the first watering with Cd-containing nutrient solution. The concentrations applied were 2%, 0.5 mM, or 0.24 g Sm L^−1^ of MEg, respectively. Two more foliar sprays were performed one week and two weeks later. Depending on our initial study, the concentrations of MEg and Sm used for this study were also nominated, as these concentrations conferred the best preferable responses (Table S1). To increase the penetration efficiency of the spray solution, Tween-20 was used a few drops at a time as a surfactant. The Optima 3300DV ICP-MS instrument (Perkin-Elmer, Inductively Coupled Plasma, Waltham, Mass Spectrometer, MA 02451, USA) was utilized to maintain the Cd concentration at 0.5 mM by continuous measurement. The trials were terminated one month after the first Cd application. Eight treatments were applied for this study and are presented in Table 2. A completely randomized design (CRD) was used to arrange the experimental pots.

### 2.2. Preparations of Maize Grain Extract (MEg) and Silymarin (Sm) Solutions

The full method outlined in [3,23] was utilized to prepare an extract from maize grain (MEg) selecting the local genotype of Egyptian *Zea mays*. The grains were covered with wet cotton and a clean piece of cloth and kept until soft and then transferred for milling using enough distilled water. Then, the mixture was filtered under a vacuum. Black bottles were used to keep the filtrate undercooling (4 °C). The remaining residue was utilized to obtain an alcoholic extract by extraction with methanol (70%) for 3 days. Again, filtration was performed. Using a rotary evaporator, the filtrate was evaporated until the alcohol was completely removed. Both extracts (e.g., the alcoholic and aqueous) were mixed well; then, the mixture was concentrated to reach the target concentrations. Note: It is preferable to use the extract immediately, otherwise it will be kept under freezing (−20 °C) until use.

Some major ingredients in MEg were detected. For free proline [27], ascorbate [28], and glutathione [29], the contents were determined in MEg, and Sm content was then determined according to the methods detailed in [30,31]. DPPH-radical scavenging activity was assayed to specify the antioxidative activity in MEg using the 1,1-diphenyl-2-picrylhydrazyl [32]. The fresh extract was used for endogenous levels of phytohormones (e.g., auxins, gibberellins, cytokinins, including zeatin-type-cytokinins). The sample was frozen in liquid N for preparing to extract different phytohormones that were then analyzed using the GC/MS system [33]. The results of all assessments are shown in Table 3. As shown, the content of Sm detected in MEg was low (0.02 µg g^−1^ FW), and thus MEg at 2% level was enriched with Sm by adding it at a concentration of 0.5 mM (i.e., 0.24 g Sm L^−1^ of MEg).

### 2.3. Maize Morphological Traits

A large graduated ruler was utilized to record plant height (from the surface level of the soil to the end of the first upper leaf in cm). After counting the leaves on each plant, all green leaves per plant were scanned utilizing a Stationary Leaf Area Meter LI-3100C (LI-COR, Lincoln, NE, USA) to record the total leaves area per plant. The shoot of each plant was weighed to record its fresh weight, and after oven-drying at 70 °C, the shoot dry weight was recorded per plant after at least two constant dry weights.

### 2.4. Leaf Photosynthetic Efficiency

Using the first fully expanding upper leaf on each plant, detailed procedures in [34,35,36] were applied for assessing the instantaneous efficiency of carboxylation (iEC; µmol m^−^^2^ s^−^^1^), photosynthetic pigment contents (mg g^−^^1^ FW), and photochemical activity (using potassium cyanide technique), respectively.

The fluorescence measurements of chlorophyll *a* were made utilizing a modulated fluorometer (PAM-2000, Heinz-Walz). The measurements were made using the saturation pulse method [34] on leaves preadapted to the dark in a growth chamber (12 h at 28 °C and 70% relative humidity). The potential quantum efficiency of photosystem II (FSII) *F_v_*/*F_m_* (*F_v_* is the variable fluorescence (*F_v_* = *F_m_* − *F*_0_), *F*_0_ is the initial fluorescence, and *F_m_* is the maximum fluorescence values) was measured. After fluorescence measurements, the plants remained in the growth chamber for two hours, under the following conditions: 28 °C, 350 µmol m^−2^ s^−1^ of flow density photosynthetically active photons (DFFFA), 70% relative humidity. Then, the measures of net CO_2_ assimilation rate and internal concentration of CO_2_ with a system of portable photosynthesis (LCi, ADC) in sheets submitted to DFFFA of 900 µmol.m^−2^·s^−1^ supplied by a halogen lamp. The iEC was calculated as follows [34]:

iEC = assimilation rate/internal concentration of CO_2_ (1)

Total chlorophyll and carotenoids were extracted with dimethyl sulfoxide and the absorbances were read at 480, 649, and 665 nm utilizing a Mullikan GO plate reader (Thermo Fisher Scientific; Waltham, MA, USA) [35]. For photochemical activity, chloroplast fragments from *Zea mays* were prepared and stored in a potassium chloride–sucrose medium. Fragments were incubated at 2 °C in sealed tubes at pH 6.8 in the presence of different concentrations of potassium cyanide. At intervals, 1 mL samples were removed and the Hill reaction activity was measured at 10 °C using a potentiometric method [36].

### 2.5. Oxidative Stress Biomarker Levels and Their Damage in Maize Plants

Using the first fully expanding upper leaf on each plant, the detailed procedures in [37,38,39] were applied for assessing the levels of O_2_^•−^ (A_580_ g^−1^ FW), H_2_O_2_, and MDA (µmol g^−1^ FW), respectively.

For determining O_2_^•^^−^, the content (µmol g^−1^ FW) was evaluated using sample fragments (1 × 1 mm, 0.1 g) that flooded using a 10 mM K-phosphate buffer (pH 7.8); each of them were mixed with each of NBT (0.05%) and NaN_3_ (10 mM) for 1 h at room temperature. The mixture was heated for 0.25 h at 85 °C. The mixture was then cooled rapidly. The absorbance readings were taken at 580 nm [37]. For determining H2O2 level (µmol per g of leaf FW), 0.25 g fresh leaf was homogenized in 5 mL 5% TCA. Homogenate centrifugation (12,000× *g*) was performed at 4 °C for 15 min. After collecting the supernatant, it was added to a reaction medium, 10 mM of buffer (potassium phosphate, pH 7.0) + 1 M of KI. Using a spectrophotometer, the absorbance reading was recorded at 390 nm using a standard prepared from H_2_O_2_ [38]. Peroxidation of lipids was assessed by determining the level of malondialdehyde (MDA; µmol per g of leaf FW). MDA assessment was performed using the same H_2_O_2_ extracts. The calculation was performed with 0.155 × 10^−3^ M^−1^ cm^−1^ as a coefficient of molar extinction to record the content of MDA [39].

Total ions seeped from leafy tissue were measured depending on the method depicted in [40]. Electrical conductivities (EC_1_, EC_2_, and EC_3_) of 20-leafy tissue disc solution were recorded three times—pre-heating, after 30-min heating at 45−55 °C, and after 10 min of boiling (100 °C), respectively. Using a known formula, EL was computed:

EL (%) = [(EC_2_ − EC_1_)/EC_3_] × 100 (2)

### 2.6. Determination of Cd Content

Using the first fully expanding upper leaf on each plant, Cd content (mg kg^−1^ leaf dry weight) was analyzed using an atomic absorption spectrophotometer (Perkin-Elmer, Model 3300) [41]. After drying, a sample of 0.1 g was digested using an acidic mixture (e.g., 2 mL perchloric acid, 80% + 10 mL H_2_SO_4_, concentrated) for 12 h. After dilution of the digested sample to reach 100 mL with distilled water, Cd^2+^ was measured.

### 2.7. Determination of Antioxidant Contents and Redox State

Using the first fully expanding upper leaf on each plant, the methods detailed in [27,28,29] were practiced for assessing proline content (µmol g^−1^ DW), AsA content (µmol g^−1^ FW) and AsA redox state, and GSH content (µmol g^−1^ FW) and GSH redox state, respectively.

The method detailed by Bates et al. [27] was followed to determine the leaf content of free proline. After extraction of 0.5 g of fresh leaf sample using 3%, *v*/*v*, sulphosalicylic acid and centrifugation at 10,000× *g* for 10 min, 2 mL supernatant was mixed with 2 mL solution of freshly prepared acid ninhydrin. The mixture was incubated in a water bath at 90 °C for 30 min. The reaction was terminated in an ice bath and the mixture was then extracted again by mixing with 5 mL toluene. The mixture was separated in the dark for 20 min at room temperature. The toluene phase was collected and its absorbance was read at 520 nm.

The Kampfenkel and Van Montagu [28] method was applied to estimate AsA level (µmol per g of leaf FW). The mixture of 30 mM of buffer (potassium phosphate, pH 7.4) + TCA (2.5%) + phosphoric acid (8.4%) + bipyridyl (0.8%) + ferric chloride (0.3%) was received leaf extract. The reaction was conducted (40 °C, 30 min), and absorbance was read at 525 nm. Content of AsA + DHA (oxidized AsA) was assessed after the addition of extract to 500 µM of DTT to estimate total AsA reduction by reading the absorbance on 525 nm and L-AsA was used as a standard, and the following formula was used to record AsA redox state:

AsA redox state (%) = [AsA/(AsA + DHA)] × 100(3)

The Griffith [29] method was applied to assess the levels (µmol per g of leaf FW) of reduced GSH and the total (GSH + GSSG). For GSH assessment, extract of leaf + 0.13 M of buffer (sodium phosphate, pH 7.4) + 0.007 M of buffer (sodium phosphate, pH 6.8) + 0.006 M of 5,5-dithiobis-(2-nitrobenzoic acid) (DTNB) as a reaction mixture was stayed on 30 °C for 10 min. Then, absorbance reading was taken on 412 nm. Total GSH level was determined after reducing GSSG to GSH by adding leaf extract to 0.13 M of buffer (sodium phosphate, pH 7.4) + 1 U of GSH-reductase. The absorbance was read on 412 nm. Content of GSH, as well as GSH+GSSG, was assessed along with a standard (GSH), and calculation of GSH redox state was done:

GSH redox state % = [GSH/(GSH + GSSG)] × 100(4)

### 2.8. Determination of Silymarin (Sm) Content

The Sm content was determined as detailed in [30,31]. The leaf sample (the first fully expanding upper leaf on each plant) was extracted for Sm using a Soxhlet apparatus. For extraction, methanol (200 mL) was used and the extract was then evaporated to dryness. The resulted sample was reconstituted in HPLC grade methanol (25 mL), and the reconstituted sample was diluted with methanol to assess Sm content (μg g^−1^ DW) using the HP 1100 Liquid Chromatograph (Thermo Fisher Scientific; Waltham, MA, USA).

### 2.9. Phytohormone Analysis

Using the first fully expanding upper leaf on each plant, the level of phytohormones was assessed using the HPLC apparatus. After extraction and centrifugation of each leaf sample, leaf debris was found between two formed phases. After concentration and resolubilization of the lower layer, injection into the apparatus column was performed for analysis. The separation was carried out for IAA, GA_1_, GA_3_, and trans-zeatin using MeOH [33].

### 2.10. Enzymatic Antioxidant Activities Assaying and Molecular Study

In an ice bath, 500 mg of fresh leaf tissue was pulverized while using 10 mL of 50 mM K-phosphate buffer (K_2_HPO_4_ + KH_2_PO_4_, Merck, Germany, pH 7.8). Centrifugation was practiced for the mixture at 10,000 × *g* at 4 °C for 15 min. The extract protein concentration was determined based on the method of Bradford [42]. SOD (EC 1.15.1.1) activity assay was done as detailed in Kono’s [43] method. Sodium carbonate (Na_2_CO_3_; a buffer) and nitroblue tetrazolium (NBT; a substrate) were used and the inhibition in the rate of NBT reduction was read at 540 nm. CAT (EC 1.11.1.6) activity assay was done as described in Aebi [44] method. Potassium phosphate (KH2PO4; a buffer) and hydrogen peroxide (H_2_O_2_; a substrate) were used, and changes in the absorbance were read at 240 nm. An APX (EC 1.11.1.11) activity assay was performed based on the method of Rao et al. [45] and the absorbance was read at 290 nm. A GR (EC 1.6.4.1) activity assay was performed and the NADPH oxidation was monitored for three absorbance readings recorded at 340 nm [45].

Using the first fully expanding upper leaf on each plant, mRNA levels were assessed, and isolation of total RNA from leaf sample was performed using an RNeasy Mini Kit (Qiagen GmbH, Germany). Using RevertAid H Minus First Strand cDNA Synthesis Kit (Fermentas GmbH, Germany), the subsequent synthesis of cDNA was performed. Primer sequences for semiquantitative and quantitative RT-PCR of the stress-related genes in *Zea mays* are presented in Table 4. The analysis for qRT–PCR was implemented on the iCycler Thermal Cycler (Bio-Rad, USA) using the instructions of iQ SYBR Green Supermix (Bio-Rad, USA) manufacture. As a reference gene for qPCR data normalization, the actin gene was used. Using LinRegPCR Software, the efficiency of the reactions was calculated [46]. Using the equation depicted in [47], signal values were derived from threshold cycles, with the average background subtracted.

### 2.11. Analysis of the Resulting Data

The data resulted from this study were analyzed by applying one-way analysis of variance [48]. For this purpose, the statistical software Statistix^®^, version 8.1 (Copyright 2005, Analytical Software, USA) was applied. Treatment means were compared utilizing the LSD Test at *P* ≤ 0.05.

## 3. Results

### 3.1. The Desired Characteristics of Maize Grain Extract (MEg) Used in This Study

Table 5 shows the main characteristics of MEg derived from maize grains. In addition to having high antioxidative activity (89.22%), MEg is rich in antioxidants (e.g., proline, ascorbate, and glutathione) and phytohormones (IAA, GA_1_, GA_3_, cytokinins, including trans-zeatin, and salicylic acid (SA)), but it is poor in Sm compared to those in maize leaves. These essential ingredients make MEg a valuable biostimulator. Therefore, we used this valuable extract either alone or after enriching it with Sm to assess its defensive effects on Cd-stressed maize plants.

### 3.2. The Response of Maize Plant Morphology and Leaf Photosynthetic Efficiency to MEg and/or Sm

**Figure 1:**
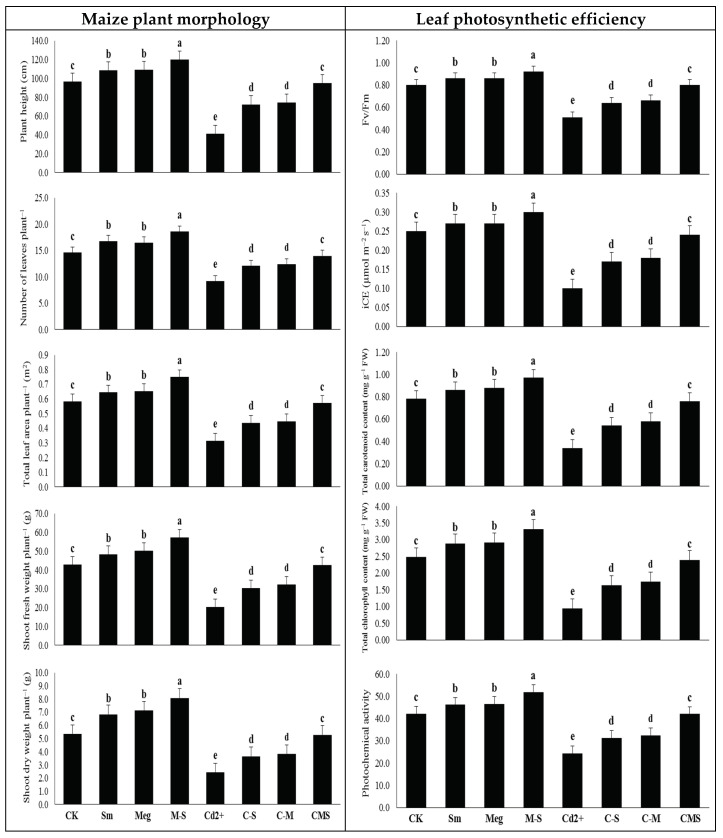
Response of maize plant morphology and leaf photosynthetic efficiency (carboxylation efficiency (iCE), PSII efficiency (*F_v_*/*F_m_*), total chlorophyll and carotenoid contents, and photochemical activity) to foliar application of silymarin (Sm), maize grain extract (MEg), or silymarin-enriched maize grain extract (MEg-Sm) under Cd stress. The same letters on the bars (mean ± SE) of the parameters indicate nonsignificant differences, whereas the significant differences are a result of different letters according to the least significant difference (LSD) test (*P* ≤ 0.05). CK = control, Sm = silymarin, Meg = maize grain extract, M-S = silymarin-enriched maize grain extract, C-S = Cd+Sm, C-M = Cd+Meg, CMS = Cd+Meg+Sm.

In the absence of stress, plant height, leaves number, leaves area per plant, shoot fresh weight, shoot dry weight, and leaf photosynthetic efficiency (in terms of carboxylation efficiency (iCE), PSII efficiency (Fv/Fm), total chlorophyll and carotenoid contents, and photochemical activity) were increased significantly with 2% MEg or 0.5 mM Sm. MEg enriched with Sm (MEg-Sm) was more efficient, increasing plant height by 24.3%, leaves number by 27.4%, leaves area by 28.3%, shoot fresh weight by 33.6%, shoot dry weight by 50.7%, iCE by 130.1%, Fv/Fm by 52.2%, chlorophyll content by 82.2%, carotenoid content by 108.3%, and photochemical activity by 116.8% compared to the corresponding (normal) control (Figure 1).

Plant height, leaves number, leaves area per plant, shoot fresh weight, shoot dry weight, iCE, *F_v_*/*F_m_*, total chlorophyll content, total carotenoid content, and photochemical activity were markedly declined with 0.5 mM Cd applied in the irrigation solution. The decreases were 57.3%, 37.0%, 46.2%, 52.3%, 54.5%, 60.0%, 36.3%, 62.1%, 56.4%, and 42.3%, respectively, compared to the normal control (Figure 1).

Compared to the stressed control (0.5 mM Cd), all of the above parameters were significantly increased with MEg or Sm, whereas MEg-Sm was more efficient, as all ofthe above parameters increased by 130.1%, 52.2%, 82.2%, 108.3%, 116.8%, 140.0%, 56.9%, 154.3%, 123.5%, and 72.8%, respectively. MEg-Sm treatment improved the morphological parameters and leaf photosynthesis efficiency of Cd-stressed plants to reach the same level as normal control plants (Figure 1).

### 3.3. The Response of Oxidative Stress Markers and Their Damages to MEg and/or Sm

**Figure 2:**
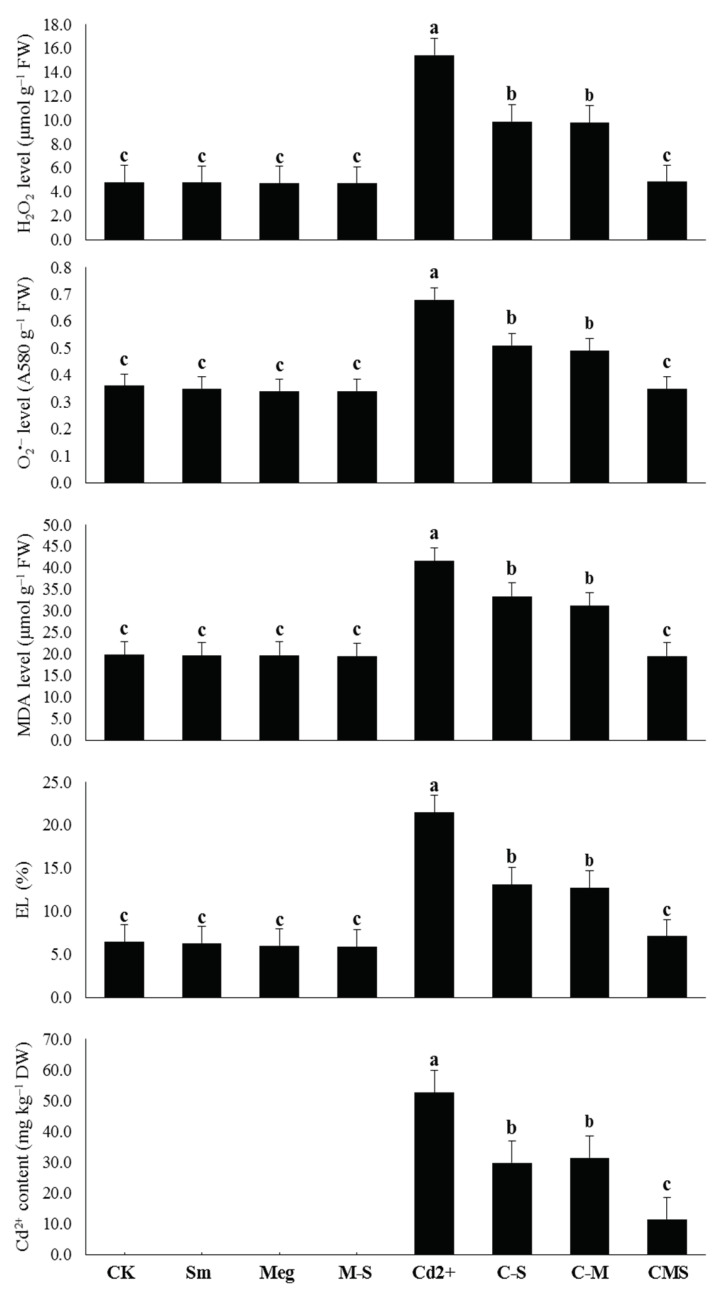
Response of oxidative stress markers (superoxide (O2^•−^) and hydrogen peroxide (H2O2) levels) and their damage (lipid peroxidation as malondialdehyde (MDA) level and ionic leakage (EL, %)) and Cd content of maize plants to foliar application of silymarin (Sm), maize grain extract (MEg), or silymarin-enriched maize grain extract (MEg-Sm) under Cd stress. The same letters on the bars (mean ± SE) of the parameters indicate nonsignificant differences, whereas the significant differences are a result of different letters according to the least significant difference (LSD) test (*P* ≤ 0.05). (*P* ≤ 0.05). CK = control, Sm = silymarin, Meg = maize grain extract, M-S = silymarin-enriched maize grain extract, C-S = Cd+Sm, C-M = Cd+Meg, CMS = Cd+Meg+Sm.

In stress-free conditions, levels of O_2_^•^^−^, H_2_O_2_, MDA, and EL were slightly decreased, whereas Cd was not detected with 2% MEg, 0.5 mM Sm, or even with MEg-Sm, which was more efficient, compared to the corresponding (normal) control (Figure 2).

The levels of O_2_^•^^−^, H_2_O_2_, MDA, EL, and Cd were considerably elevated with the addition of 0.5 mM Cd in the irrigation solution. The unwanted increases were 88.9%, 219.9%, 110.1%, 233.2%, and 52.6%, respectively, compared to the normal control (Figure 2).

Compared to the stressed control (0.5 mM Cd), all of the above parameters were significantly decreased with MEg or Sm; however, MEg-Sm was more efficient, with all of the above parameters decreasing by 48.5%, 68.6%, 53.1%, 67.0%, and 78.3%, respectively. Cd-stressed plants were able to minimize markers of oxidative stress, which was reflected in the considerable reduction of El and Cd^2+^ levels upon receiving MEg-Sm as a foliar spray (Figure 2).

### 3.4. The Response of Free Proline Content, Levels, and Redox States of Ascorbate (AsA) and Glutathione (GSH) to MEg and/or Sm

**Figure 3:**
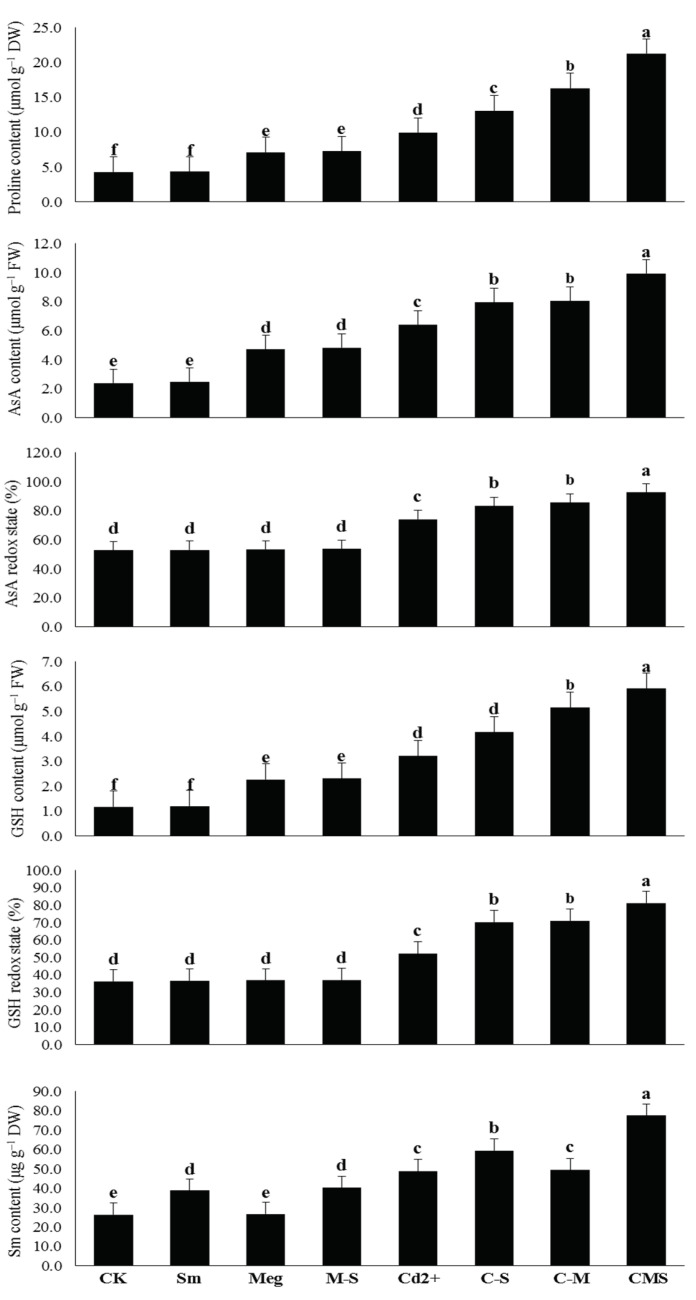
Response of free proline content, level, and redox state of ascorbate (AsA) and glutathione (GSH) of maize plants to foliar application of silymarin (Sm), maize grain extract (MEg), or silymarin-enriched maize grain extract (MEg-Sm) under Cd stress. The same letters on the bars (mean ± SE) of the parameters indicate nonsignificant differences, while the significant differences are a result of different letters according to the least significant difference (LSD) test (*P* ≤ 0.05). CK = control, Sm = silymarin, Meg = maize grain extract, M-S = silymarin-enriched maize grain extract, C-S = Cd+Sm, C-M = Cd+Meg, CMS = Cd+Meg+Sm.

Under normal conditions, the contents of proline, AsA, GSH, and Sm were increased as the redox state of AsA and GSH increased by 2% MEg or 0.5 mM Sm; the increase in Sm content was significant with Sm treatment, whereas the increase in proline, AsA, and GSH content was significant with MEg treatment. MEg-Sm was more efficient; it significantly increased proline content by 69.0%, AsA content by 100.8%, AsA redox state by 1.7%, GSH content by 96.6%, GSH redox state by 1.9%, and Sm content by 52.7% compared to the corresponding (normal) control (Figure 3).

Proline, AsA, GSH, and Sm levels, as well as the redox state of AsA and GSH, were significantly elevated under Cd stress. The increases were 130.6%, 167.2%, 172.9%, 84.8%, 40.5%, and 43.7%, respectively, compared to the normal control (Figure 3).

Compared to the stressed control (0.5 mM Cd), all of the above parameters were further increased with MEg or Sm. However, MEg-Sm was more efficient, with all of the above parameters increasing by 113.7%, 54.3%, 83.9%, 59.0%, 24.8%, and 55.6%, respectively. The Cd-stressed maize plants were able to amass more antioxidants such as Proline, AsA, GSH, and Sm to efficiently cope with markers of oxidative stress upon receiving MEg-Sm as a foliar spray (Figure 3).

### 3.5. The Response of Enzyme Activities and Hormonal Levels to MEg and/or Sm

**Figure 4:**
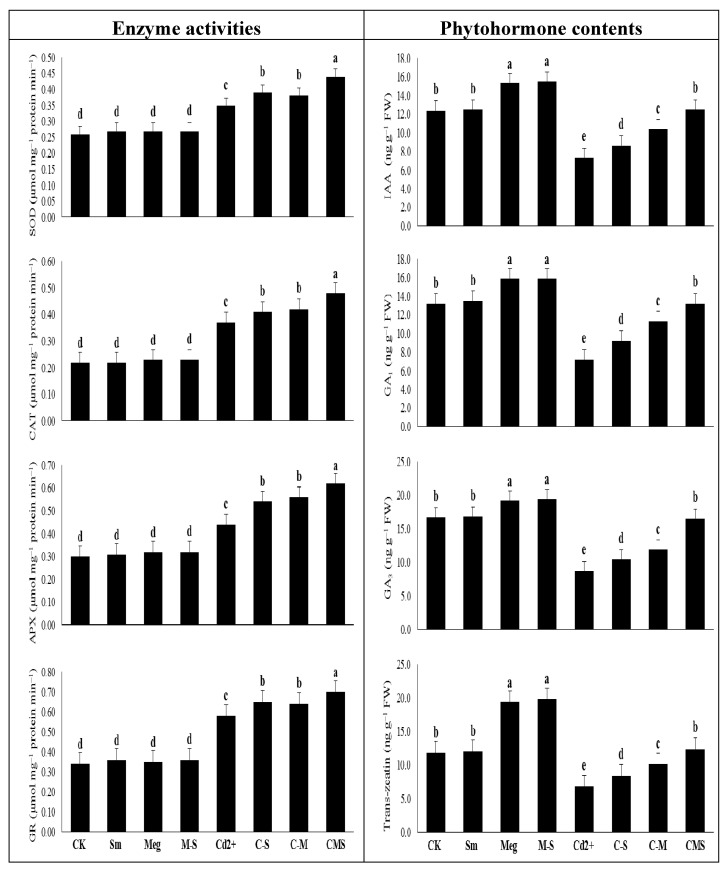
Response of enzyme (superoxide dismutase (SOD), catalase (CAT), ascorbate peroxidase (APX), and glutathione reductase (GR)) activities, as well as leaf phytohormone levels (indole-3-acetic acid (IAA), gibberellic acids 1 and 3 (GA1 and GA3), and cytokinin (trans-zeatin)) of maize plants to foliar application of silymarin (Sm), maize grain extract (MEg), or silymarin-enriched maize grain extract (MEg-Sm) under Cd stress. The same letters on the bars (mean ± SE) of the parameters indicate nonsignificant differences, whereas the significant differences are a result of different letters according to the least significant difference (LSD) test (*P* ≤ 0.05). CK = control, Sm = silymarin, Meg = maize grain extract, M-S = silymarin-enriched maize grain extract, C-S = Cd+Sm, C-M = Cd+Meg, CMS = Cd+Meg+Sm.

In the nonstress conditions, SOD, CAT, APX, and GR activities were slightly elevated with MEg, Sm, or even MEg-Sm. However, levels of IAA, GA_1_, GA_3_, and trans-zeatin were slightly increased with Sm but significantly increased with MEg or MEg-Sm, with a nonsignificant preference for MEg-Sm treatment, which increased SOD, CAT, APX, and GR activities by 3.8%, 4.5%, 6.7%, and 5.9%, respectively, and IAA, GA_1_, GA_3_, and trans-zeatin levels by 25.0%, 20.5%, 16.2%, and 67.8%, respectively, as compared to the corresponding (normal) control (Figure 4).

Under Cd stress conditions, SOD, CAT, APX, and GR activities were markedly elevated by 34.6%, 68.2%, 46.7%, and 70.6% respectively, whereas the levels of IAA, GA_1_, GA_3_, and trans-zeatin were significantly decreased by 41.1%, 45.5%, 47.9%, and 42.4%, respectively, compared to the normal control (Figure 4).

Compared with the Cd-stressed control, SOD, CAT, APX, and GR activities were significantly increased along with increased levels of IAA, GA_1_, GA_3_, and trans-zeatin with MEg or Sm. However, MEg-Sm was more efficient, with all of the above parameters increasing by 25.7%, 29.7%, 40.9%, and 20.7% for enzyme activities and by 71.2%, 83.3%, 89.7%, and 82.4% for hormonal levels, respectively. Cd-stressed maize plants were able to increase their enzymatic activities along with various antioxidants to cope with markers of oxidative stress and stabilize their hormonal contents upon receiving MEg-Sm as a foliar spray (Figure 4).

### 3.6. The Response of Gene Transcript Levels to MEg and/or Sm

**Figure 5:**
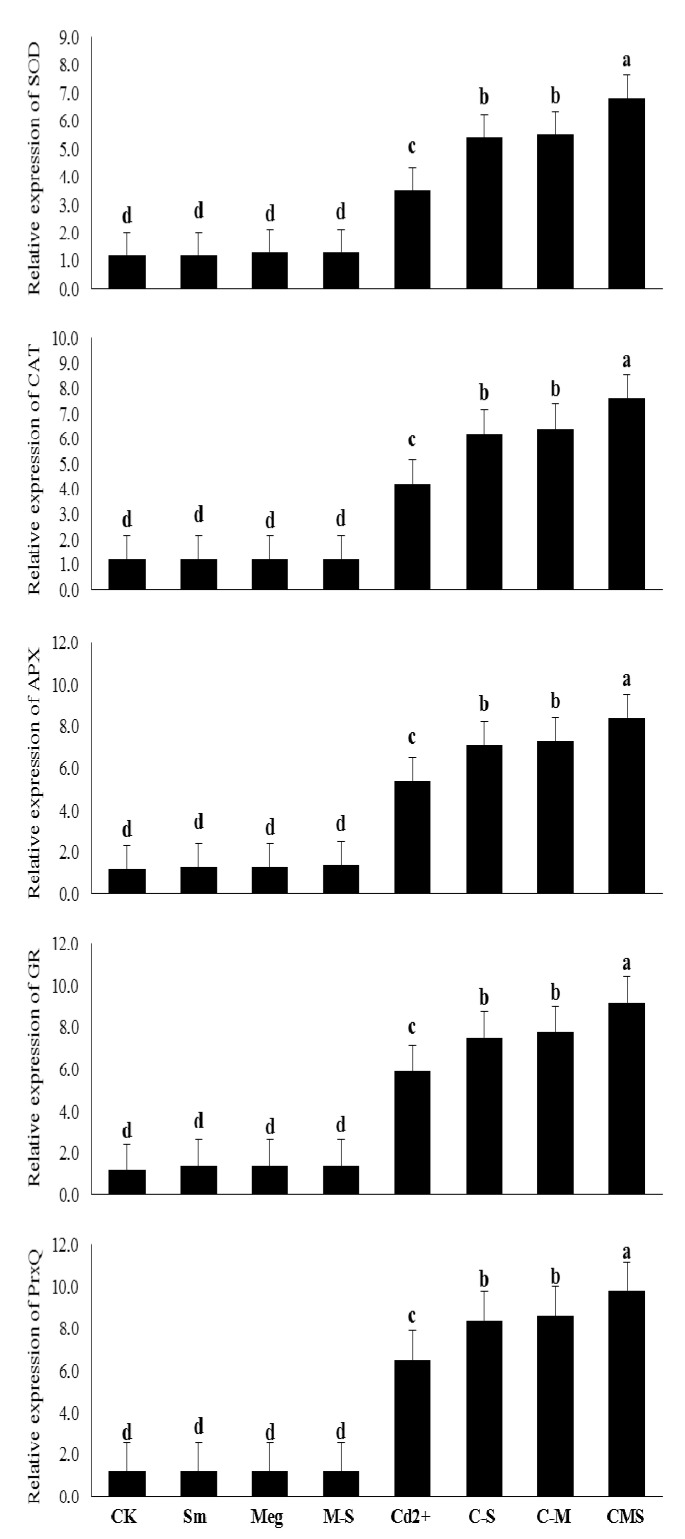
Response of antioxidant enzyme-encoding gene transcript levels (transcriptional levels were quantified by qPCR relative to actin transcriptional level, qPCR data represent the average from three independent experiments with two technical replicates each) of maize plants to foliar application of silymarin (Sm), maize grain extract (MEg), or silymarin-enriched maize grain extract (MEg-Sm) under Cd stress. The same letters on the bars (mean ± SE) of the parameters indicate nonsignificant differences, whereas the significant differences are a result of different letters according to the least significant difference (LSD) test (*P* ≤ 0.05). CK = control, Sm = silymarin, Meg = maize grain extract, M-S = silymarin-enriched maize grain extract, C-S = Cd+Sm, C-M = Cd+Meg, CMS = Cd+Meg+Sm.

Using quantitative and conventional RT-PCR, genetic relative expression of enzymatic antioxidants (SOD, CAT, APX, GR, and PrxQ) were signalized for normal and Cd-stressed maize plants and stressed plants treated with MEg, Sm, or MEg-Sm (Figure 5).

Under normal conditions, transcriptional levels of SOD, CAT, APX, GR, and PrxQ genes were slightly or not affected by MEg, Sm, or even MEg-Sm. The recorded increases of gene expression levels were 8.3%, 0%, 16.7%, 16.7%, and 0%, respectively, upon treatment by MEg-Sm compared to the corresponding (normal) control (Figure 5).

Transcriptional levels of SOD, CAT, APX, GR, and PrxQ genes were markedly increased under 0.5 mM Cd, and the increases were 191.7%, 250.0%, 350.0%, 391.7%, and 441.7%, respectively, compared to the normal control (Figure 5).

Compared to the Cd-stressed control, the transcript levels of the SOD, CAT, APX, GR, and PrxQ genes were significantly increased with MEg or Sm, whereas MEg-Sm was more efficient, and increased the aforementioned gene transcriptional levels by 94.3%, 81.0%, 55.6%, 55.9%, and 50.8%, respectively. Cd-stressed maize plants were able to transcribe more gene-encoding enzymatic antioxidants along with various nonenzymatic antioxidants to cope with markers of oxidative stress upon receiving MEg-Sm as a foliar spray (Figure 5).

### 3.7. The Interrelationship among the Traits Evaluated in Response to MEg and/or Sm

**Figure 6:**
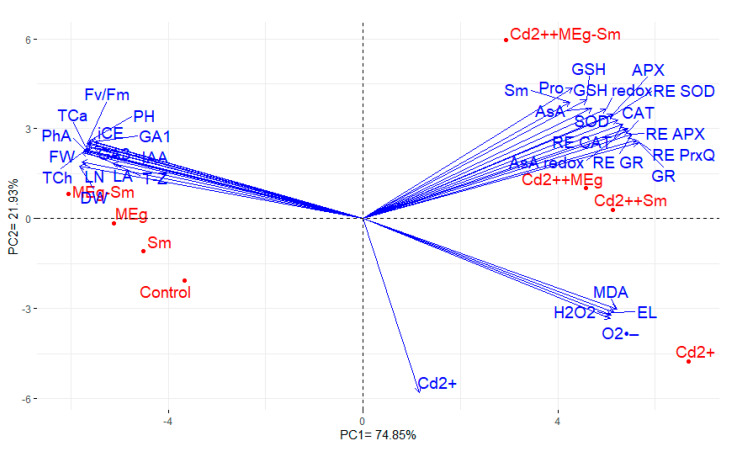
Biplot of analysis of prime components demonstrating the relationship among the traits assessed. Plant height (PH), number of leaves (LN), total leaf area (LA), shoot fresh weight (FW), shoot dry weight (DW), instantaneous carboxylation efficiency (iCE), photosystem II efficiency (Fv/Fm), total chlorophyll (TCh), carotenoid contents ((TCa), photochemical activity (PhA), superoxide (O2^•−^), hydrogen peroxide (H2O2), lipid peroxidation as malondialdehyde (MDA), ionic leakage (EL), cadmium (Cd2+), proline (Pro), ascorbate (AsA), glutathione (GSH), ascorbate redox state (AsA redox), glutathione redox state (GSH redox), silymarin (Sm), superoxide dismutase (SOD), catalase (CAT), ascorbate peroxidase (APX), glutathione reductase (GR), relative expression of SOD (RE SOD), relative expression of CAT (RE CAT), relative expression of APX (RE APX), relative expression of GR (RE GR), relative expression of PrxQ (RE PrxQ), indole-3-acetic acid (IAA), gibberellic acids 1 and 3 (GA1 and GA3), and cytokinin trans-zeatin (T-Z).

Key components were computed to verify the association among the attributes evaluated. The first two primary components displayed the most variability by approximately 96.8% (74.9% by PC1 and 21.9% by PC2). Next, PC1 and PC2 were utilized to construct the PC-biplot (Figure 6). Parallel or close vectors represented features, indicating a strong positive association, whereas vectors with angles close to 180° indicated a negative association. The traits evaluated in the current study can be divided into three sets. The first set consisted of Pro, AsA, GSH, AsA redox state, GSH redox state, Sm, SOD, CAT, APX, GR, RE SOD, RE CAT, RE APX, RE GR, and RE PrxQ. The second set included PH, LN, LA, FW, DW, iCE, Fv/Fm, TCh, TCa, PhA, IAA, GA_1_, GA_3_, and T-Z. The third set contained Cd^2+^, MDA, EL, O_2_^•−^, and H_2_O_2_. The traits within each group displayed a high association with each other, whereas an intermediate positive association was detected among traits of the first and third groups. The first group exhibited a negative association with the second group.

## 4. Discussion

No information is available on the foliar application of maize plants with a maize grain extract (MEg) enriched with silymarin (Sm); MEg-Sm to attenuate the adverse impacts of cadmium (Cd) stress on plant performance, which responded positively to the novel MEg-Sm. Recently, some articles have been reported positive changes in plant growth, biochemical attributes, and plant defensive system (e.g., antioxidative enzymes and nonenzymatic antioxidants) after treating plants with MEg alone under some stresses [3,20,21,22,23], indicating the importance of MEg. However, the current study provides impressive results such as a considerable increase in maize plant performance and defense system along with gene expression related to antioxidant enzymes due to the application of MEg-Sm, which outperformed MEg alone due to that Sm increased the efficiency of the extract.

Presently, sustainable maize returns challenge various issues such as reduced soil fertility and increased degradation due to contamination of farmland with heavy metals, including Cd. Cd stress is one of the key concerns facing maize production, restricting crop yield. Cd frequently causes disturbances of various morphophysiobiochemical and molecular features. It restricts plant growth, disrupts chlorophyll biosynthesis, and thus photosynthesis, and negatively affects the defense system and antioxidant gene expression in plants [8,14]. Therefore, attenuating the toxic effect of Cd on plant growth and biochemical processes remains a constant concern of scientists. There is an urgent need to ameliorate Cd tolerance in maize through sustainable and ecofriendly strategies, which are the key to achieving security for more foods for an ever-expanding population. In the present study, 0.5 mM Cd severely decreased maize plant growth traits (e.g., plant height, leaves number, leaves area per plant, fresh and dry weight of shoot system), photosynthetic efficiency (instantaneous carboxylation efficiency; iCE, pigment contents, *F_v_*/*F_m_*, and photochemical activity) and hormonal contents (Figure 1 and Figure 4, Table 6). These negative results coincided with a harmful increase in markers of oxidative stress levels (O_2_^•^^−^ and H_2_O_2_), lipid peroxidation (MDA), ionic leakage (EL), and accumulation of Cd (Figure 2). All of these negative results encouraged an increase in the plant’s enzymatic and nonenzymatic antioxidative defense system and Sm content (Figure 3 and Figure 4, Table 6), and transcriptional level of genes related to enzymatic antioxidants (Figure 5, Table 6) to enable plants to cope with markers of oxidative stress overproduced by Cd stress.

The number of green leaves obtained from the tallest plants should be optimized; thus, green leaf area is a pivotal strategy for increasing photosynthesis efficiency and increasing dry matter output. Under normal or Cd stress conditions, foliar treating maize plants with MEg or Sm led to a significant rise in plant height, leaves number, and leaves area, which reflected positively on plant weight, especially dry matter output (Figure 1, Table 6). Treatment with Sm-enriched MEg (MEg-Sm) outperformed either MEg or Sm alone. This may be due to the improving effect of Sm, which has added to the various benefits of MEg. In addition to having a high antioxidative activity (89.22%), MEg contains several stimulating mechanisms such as antioxidants (proline, ascorbate; AsA, and glutathione; GSH) and various phytohormones (IAA, GA_1_, GA_3_, cytokinins including trans-zeatin, and salicylic acid; SA) (Table 5). The increase in plant height and number of leaves resulting from the application of MEg-Sm contributed to an increase in plant leaf area, accompanied by an increase in photosynthetic pigment contents, all of which contributed to an increase in photosynthetic efficiency (*F_v_*/*F_m_* and photochemical activity). These positive results were positively reflected in dry matter accumulation (Figure 1, Table 6). All these positive results were achieved by MEg-Sm due to the minimized levels of Cd and markers of oxidative stress, which contributed to the reduction of MDA and EL (Figure 2, Table 6).

The enhanced effect of MEg-Sm (which outperformed the enhanced effect of MEg or Sm) on transcriptional gene levels related to antioxidant enzymes (SOD, CAT, APX, CR, and PrxQ) efficiently contributed to increasing levels and activities of antioxidant enzymes (Figure 4 and Figure 5, Table 6), which in turn contributed to the improvement of hormonal homeostasis (Figure 4, Table 6). The significant improvement in the antioxidant defense state of maize plants by MEg-Sm treatment contributed to the minimization of Cd^2+^ ions (Figure 2, Table 6), which resulted in the plants recovering from stress due to ROS suppression. This may enable plants to stabilize and balance their hormones so that they perform well.

It has been shown that antioxidants and hormonal homeostasis regulate plant growth and their physiobiochemical performance under Cd stress [6,23,49]. As shown in Table 5, MEg is rich in antioxidants and phytohormones, which are important mechanisms for improving growth, dry matter production, and physiobiochemical attributes, as well as the antioxidant defense system of maize plants grown under Cd stress (Figure 1, Figure 2, Figure 3, Figure 4 and Figure 5, Table 6). As a crucial mechanism that helped maize plants withstand Cd stress, increases in AsA and GSH contents and redox states were noticed (Figure 3, Table 6). An increase in hormonal content and homeostasis was also observed as another effective mechanism that enabled plants to withstand the effects of Cd stress (Figure 4, Table 6).

The major mechanism in this regard, the observed increases in transcriptional levels of genes related to the examined antioxidant enzymes, had a major role in withstanding the adverse effects of stress [8,50] in maize plants. In addition to these key mechanisms, increases in proline and Sm contents (Figure 3, Table 6) likely contributed to the increased defenses of the maize plant against Cd stress. Since many plant growth stimuli (e.g., AsA, GSH, IAA, GA_1_, GA_3_, cytokinins including trans-zeatin, SA, proline, and Sm) are present in MEg-Sm (Table 5), which was the best treatment, it is considered a potent biostimulator to grow maize plants effectively under Cd stress (Figure 1, Figure 2, Figure 3, Figure 4 and Figure 5, Table 6).

Among the major growth stimulators present in MEg-Sm, proline, AsA, and GSH contents were greatly increased in Cd-stressed plants and contributed to stabilizing and maintaining cell membranes against stress damage [2,5,51,52,53,54,55,56,57,58,59]. These positive effects of these antioxidants might be attributed to their roles in minimizing the Cd content or other toxic elements and the levels of oxidative stress markers (O_2_^•^^−^ and H_2_O_2_; Figure 2, Table 6), thus minimizing lipid peroxidation in cell membranes. As a result of all these positive results, the photosynthetic efficiency including *F_v_*/*F_m_* and iCE was maintained (Figure 1, Table 6) due to the integrity of cellular water content [2,52,53,55]. These positive results examined in this study were positively reflected in maize plant growth and dry matter accumulation (Figure 1, Table 6). Our findings are consistent with those in [2,5,51,52,53,54,55,56,57,58,59]. Restoration of growth and dry matter accumulation of Cd-stressed maize plants are also due to the positive impacts of proline, AsA, and GSH present in MEg-Sm on increasing hormones (e.g., IAA, GA_1_, GA_3_, cytokinins including trans-zeatin, SA) content and homeostasis, which are necessary to restore the developmental growth of stressed plants [20,23,60,61,62].

Phytohormones play a large role in signaling, biochemistry, and defense pathways in plants, providing a key mechanism for relieving heavy metal stress [23,63]. The increased growth of Cd-stressed maize plants was likely related to the increased partitioning of photosynthesized substances with plant development and phytohormone levels (Figure 4, Table 6). Phytohormones regulate membrane permeability, enzyme activity, secondary metabolism, plant growth, and plant reproduction [23,64]. Elevated gibberellins (GAs) are shown in stressed plants to enhance stress tolerance by enhancing gene expression [20,65,66]. Thereafter, hormonal homeostasis under Cd stress could be a possible mechanism of GAs (e.g., GA_1_ and GA_3_) that stimulate Cd stress tolerance in the plant [65,66]. Cytokinins, including trans-Zeatin, and SA act to withstand stress. They play many regulatory roles in promoting plant growth, protein biosynthesis, and secondary metabolism [61,62,66,67]. They generally eliminate ROSs and increase the antigenicity of ABA under stress [67,68]. Another key phytohormone, IAA influences Cd toxicity, which has been relied upon to regulate many antioxidative activities, including the AsA-GSH cycle [68,69]. Altogether, phytohormones can raise antioxidant levels to reduce ROSs (e.g., O_2_^•−^ and H_2_O_2_) levels, helping reduce lipid peroxidation (MDA) to keep healthy plant growth [49]. Phytohormones eliminate stressors’ impacts and promote rates of survival [70,71] by either enhancing shoot growth or regulating processes to prohibit plant growth (e.g., dormancy, withdrawal, and aging), and thus controlling growth activities in plants [72,73]. In our investigation, elevated hormonal content by MEg, Sm, or MEg-Sm (with a high preference for MEg-Sm) (Figure 4, Table 6) was associated with higher activity of the antioxidative defense system, antioxidant gene expression (Figure 3, Figure 4 and Figure 5 and Table 6) and suppression in ROSs; O_2_^•−^ and H_2_O_2_ levels, which minimized MDA, EL, and Cd levels and maximized plant performance and photosynthesis efficiency (Figure 1 and Figure 2, Table 6).

The induced activity of antioxidant enzymes that are premium biochemical signals of stress can eliminate O_2_^•−^ and H_2_O_2_ stimulated by Cd. Despite elevated enzyme activity under Cd stress, spraying maize plants with MEg, Sm, or MEg-Sm (with a high preference for MEg-Sm) further elevated SOD, CAT, APX, and GR activity while stimulating increased transcriptional levels of the antioxidant enzyme genes (Figure 4 and Figure 5, Table 6). In this study, promotion of antioxidant gene transcript levels was associated with increased levels of proline, AsA, and GSH in contributing to increased enzymatic activities as a protective mechanism for suppressing many types of ROSs like H_2_O_2_, ^1^O_2_, O_2_^•−^, OH^−^ overproduced by stress. This positive finding contributed to keeping the metabolic processes to improve plant performance [8,23,50–59]. Given the diversity of the key bioactive ingredients present in MEg-Sm as a focal biostimulator (Table 5), it is a distinct strategy to treat maize plants as a foliar spray for the rapid growth of plants and efficient performance against Cd stress. A historical advance for controlling antioxidant genes for plant growth under stress has been reported by analyzing the transcriptional levels of antioxidant enzyme genes with the RT-qPCR technique [8,50]. Several enzyme genes are implicated in stressed plant development, and the molecular mechanisms of these antioxidant genes (e.g., SOD, CAT, APX, GR, and PrxQ) are linked with increased plant tolerance to stress [8,50]. In our study, along with the increase in nonenzymatic antioxidants, transcriptional levels of these genes increased under Cd stress and greatly increased with the application of MEg-Sm to maize plants (Figure 5, Table 6). These positive findings were reflected in the increased activity of SOD, CAT, APX, and GR enzymes (Figure 4, Table 6) to enable maize plants to effectively withstand Cd stress, increase their photosynthesis efficiency, and thus growth and dry matter accumulation (Figure 1, Table 6).

The role of silymarin, Sm (present in the MEg-Sm), as a secondary metabolite (Sm is a mixture of six flavonolignans (such as isosilybin A and B, silybin A and B, silydianin, and silychristin) and the flavonoid taxifolin), in improving plant performance under stress has not been achieved before. It has been reported that Sm can improve the productivity of plants since it accumulates in stressed plants to increase their defense systems [74,75]. This result is consistent with our results (Figure 3, Table 6). These reports [74,75] consider Sm as a powerful antioxidant, and thus its role in increasing plant resistance to stress is attributed to it as an antioxidant. Extensive studies are needed in this regard to exploring the precise mechanism of Sm for stress-tolerant plants.

The MEg-Sm results in this study are fully consistent with the characteristics of the biostimulator described by the European Biostimulant Industry Council [76] and with the findings of other work examining different stresses [3,20,21,22,23]. The growth promoters (bioactive compounds) present in MEg-Sm make it an effective biocatalyst and unique environmentally friendly strategy. MEg-Sm bioactive ingredients have functioned in this study to interplay with each other for successful plant growth under various stress conditions, including Cd stress. It has a high DPPH radical-scavenging activity (89.22%) because of its richness in various antioxidants, which possess high states of redox. This makes MEg-Sm possess pivotal mechanisms to prevent or suppress ROSs and lipid peroxidation as explained above. The complex interplaying of the bioactive ingredients of MEg-Sm occurred, in this study, to confer a robust defensive system against Cd-induced oxidative damage in favor of the performance of the maize plant.

The key ingredient biplot is a useful statistical method for assessing the interrelationship among the traits evaluated as well as the treatments examined [77,78]. In this study, the traits studied were divided into three groups. The traits exhibited a positive association with each other in the same group, whereas the traits with high positive values for PC1 displayed a negative association with those of negative PC1 values for PC1 [79,80]. Like the PC1, PC2 divided the treatments into two groups. The PC2 separated the applied treatments under Cd stress than those were performed under the absence of Cd on the other side of PC. Furthermore, the traits were identified in the first and third groups with treatments applied under Cd stress in the same sectors, indicating the importance of assessing these traits under Cd stress. These results are consistent with previous studies that showed the importance of physiological parameters as indicators under Cd stress [81,82,83,84].

## 5. Conclusions

The consequences of our study signalize an effective strategy of spraying Cd-stressed maize plants with MEg-Sm (bypassing MEg or Sm alone). This strategy can effectively promote plant growth and biomass accumulation. Enhancements of different attributes such as growth, photosynthesis efficiency, nonenzymatic antioxidants, antioxidant redox state, hormonal content and homeostasis, enzymatic antioxidants, and enzyme gene expression were focal aspects of foliar spraying of maize plants with MEg-Sm, which resulted in suppression of lipid peroxidation, ionic leakage, and oxidative damage catalyzed by ROSs (O_2_^•−^ and H_2_O_2_). These successful results were obtained as a result of the restriction of Cd ion accumulation and the activation of antioxidant defenses under Cd stress in maize plants. The interesting thing in our study is that MEg-Sm was more pronounced under Cd stress than normal conditions. The antioxidant and hormonal ingredients of MEg-Sm have functioned in our study as a natural biostimulant to interplay with each other in favor of Cd-stressed maize plants. Therefore, the findings of this study recommend the use of MEg-Sm as an effective novel biostimulator for *Zea mays* to promote various physiological and metabolic processes to boost Cd stress tolerance in maize plants. Extensive studies are needed in this regard to exploring the precise mechanism of Sm (in MEg-Sm) for stress-tolerant plants.

## Figures and Tables

**Table 1:** The nourishing solution composition prepared for watering maize seedlings.

The Chemical Substance	The chemical Formula	The Amount (µM)
Calcium nitrate	Ca(NO_3_)_2_	2000
Potassium sulfate	K_2_SO_4_	700
Magnesium sulfate	MgSO_4_	500
Monopotassium phosphate	KH_2_PO_4_	100
Potassium chloride	KCl	100
Boric acid	H_3_BO_3_	1
Manganese sulfate	MnSO_4_	1
Copper sulfate	CuSO_4_	0.25
Ammonium molybdate	(NH_4_)_6_Mo_7_O_24_	0.01
Fe–ethylenediaminetetraacetic acid (EDTA)	(OOCCH_2_)_2_NCH_2_CH_2_NCCH_2_COO)_2_FeNa·xH_2_O	100

**Table 2:** The experimental treatment description.

Treatment	Description
Control	There is no stress and no foliar applications
Sm	Foliar spray with 0.5 mM silymarin
MEg	Foliar spray with 2% maize grain extract
MEg-Sm	Foliar spray with maize grain extract enriched with silymarin (0.24 g Sm L^−1^ of MEg)
Cd^2+^	Watering the maize seedlings with a nourishing solution containing 0.5 mM Cd^2+^
Cd^2+^+Sm	Watering the maize seedlings with a nourishing solution containing 0.5 mM Cd^2+^ + foliar spray with 0.5 mM silymarin
Cd^2+^+MEg	Watering the maize seedlings with a nourishing solution containing 0.5 mM Cd^2+^ + foliar spray with 2% maize grain extract
Cd^2+^+MEg-Sm	Watering the maize seedlings with a nourishing solution containing 0.5 mM Cd^2+^ + foliar spray with maize grain extract enriched with silymarin (0.24 g Sm L^−1^ of MEg)

**Table 3:** The antioxidant and hormonal contents in maize grain extract (MEg).

Component	Unit	Value
The antioxidative compounds:		
Free proline	(µmol g^−1^ FW)	24.66 ± 0.39
Ascorbic acid (AsA)		14.26 ± 0.07
Glutathione (GSH)		8.85 ± 0.03
Silymarin (Sm)	(μg g^−1^ DW)	0.02 ± 0.00
DPPH radical-scavenging activity	%	89.22 ± 1.62
Phytohormones:		
Indole-3-acetic acid (IAA)	(μmol g^−1^ FW)	2.74 ± 0.05
Gibberellic acid 1 (GA_1_)		2.58 ±0.04
Gibberellic acid 3 (GA_3_)		2.75 ±0.06
Total cytokinins (CKs)		3.96 ± 0.08
*Trans*-Zeatin (*t*-Z)		2.55 ± 0.04
Salicylic acid (SA)		2.89 ± 0.05

Values presented in the table are means (*n* = 3 for all measures) ± standard error.

**Table 4:** Primers sequences for RT-qPCR of stress-related genes in *Zea mays.*

The Gene	Reference Seq.	5′–3′ Primer Sequence	T_A_
*Actin*	AB181991	F: CTCTGACAATTTCCCGCTCA, R: ACACGCTTCCTCATGCTATCC	58 °C
*SOD*	MG893090.1	F: TTCGCCATGCTGGTGATCTT, R: CATGGACAACTACGGCCCTT	
*CAT*	GU984379	F: GGCTGCTTGAAGTTGTTCTCCT, R: CTGCTAGTACCTCCTGATCCGTT	
*APX*	KU747079.1	F: TGGCCTGCTCTTCCTCTAGT, R: CATGCCACGCTAATCGAAGC	
*GR*	KX828561.1	F: CAACGCGCTTTGGTAACTCC, R: GGGCCCTAATGAAGTGGAGG	
*PrxQ*	AY789643	F: ACTTCACGCTCAAGGACCAG, R: CCGCCTTCTTGTACTTCTCG	

**Table 5:** Antioxidant and hormonal contents detected in maize grain extract (MEg) compared to their levels in maize leaves.

Component	Unit	Value in MEg	Value in Maize Leaf
*The antioxidative compounds:*			
Free proline	(µmol g^−1^ FW)	24.66 ± 0.39	0.54 ± 0.01
Ascorbic acid (AsA)		14.26 ± 0.07	2.41 ± 0.02
Glutathione (GSH)		8.85 ± 0.03	1.18 ± 0.02
Silymarin (Sm)	(μg g^−1^ DW)	0.02 ± 0.00	3.31 ± 0.03
DPPH radical-scavenging activity	%	89.22 ± 1.62	Not determined
*Phytohormones:*			
Indole-3-acetic acid (IAA)	(μmol g^−1^ FW)	2.74 ± 0.05	0.22 ± 0.00
Gibberellic acid 1 (GA_1_)		2.58 ±0.04	0.04 ± 0.02
Gibberellic acid 3 (GA_3_)		2.75 ±0.06	0.05 ± 0.03
Total cytokinins (CKs)		3.96 ± 0.08	Not determined
*Trans*-Zeatin (*t*-Z)		2.55 ± 0.04	0.05 ± 0.02
Salicylic acid (SA)		2.89 ± 0.05	Not determined

Values presented in the table are means (*n* = 3 for all measures) ± standard error.

**Table 6:** Changes (%) in morphophysiobiochemistry attributes, and relative expression of antioxidant enzyme genes relative to the control in Cd-stressed maize plants treated with maize grain extract enriched with silymarin. Three color scale heatmap, yellow as the midpoint of control and parameters with insignificant values compared to control, red for changes below control values, and green for changes over control values.

Parameter	Treatments							
	Control	Sm	MEg	MEg-Sm	Cd^2+^	Cd^2+^+ Sm	Cd^2+^+ MEg	Cd^2+^+MEg-Sm
Plant height	96.4 c	+12.8 b	+13.3 b	+24.3 a	−57.3 e	−24.9 d	−22.7 d	−1.7 c
Leaf number	14.6 c	+15.1 b	+13.0 b	+27.4 a	−37.0 e	−17.1 d	−15.1 d	−4.1 c
Leaf area	0.584 c	+10.3 b	+11.6 b	+28.3 a	−46.2 e	−25.3 d	−23.3 d	−2.1 c
Shoot FW	42.8 c	+13.1 b	+17.1 b	+33.6 a	−52.3 e	−29.0 d	−24.8 d	−0.7 c
Shoot DW	5.36 c	+27.6 b	+32.8 b	+50.7 a	−54.5 e	−31.7 d	−28.7 d	−1.3 c
iCE	0.25 c	+8.0 b	+8.0 b	+20.0 a	−60.0 e	−32.0 d	−28.0 d	−4.0 c
*F_v_*/*F_m_*	0.80 c	+7.5 b	+7.5 b	+15.0 a	−36.3 e	−20.0 d	−17.5 d	0 c
Chl. content	2.48 c	+16.5 b	+17.7 b	+33.9 a	−62.1 e	−33.9 d	−29.4 d	−3.6 c
Carot. content	0.78 c	+10.3 b	+12.8 b	+24.4 a	−56.4 e	−30.8 d	−25.6 d	−2.6 c
Ph.ch. activity	42.1 c	+9.7 b	+10.5 b	+23.0 a	−42.3 e	−25.9 d	−23.0 d	−0.2 c
O_2_^•^^−^ level	0.36 c	−2.8 c	−5.6 c	−5.6 c	+88.9 a	+41.7 b	+36.1 b	−2.8 c
H_2_O_2_ level	4.82 c	−1.0 c	−2.3 c	−2.7 c	+220 a	+105 b	+103 b	+0.4 c
MDA level	19.8 c	−1.0 c	−0.5 c	−2.0 c	+110 a	+68.7 b	+57.6 b	−1.5 c
EL%	6.44 c	−2.0 c	−7.1 c	−8.7 c	+233 a	+103 b	+97.2 b	+10.1 c
Pro content	4.32 f	+0.9 f	+65.3 e	+69.0 e	+131 d	+204 c	+278 b	+393 a
AsA content	2.41 e	+3.7 e	+95.9 d	+101 d	+167 c	+230 b	+234 b	+312 a
AsA redox st.	52.8 d	+0.6 d	+1.1 d	+1.7 d	+40.5 c	+57.8 b	+62.3 b	+75.4 a
GSH content	1.18 f	+2.5 f	+93.2 e	+96.6 e	+173 d	+254 c	+337 b	+402 a
GSH redox st.	36.4 d	+1.1 d	+1.4 d	+1.9 d	+43.7 c	+93.4 b	+95.6 b	+124 a
Sm content	26.4 e	+47.3 d	+1.5 e	+52.7 d	+84.8 c	+125 b	+87.1 c	+194 a
SOD activity	0.26 d	+3.8 d	+3.8 d	+3.8 d	+34.6 c	+50.0 b	+46.2 b	+69.2 a
CAT activity	0.22 d	0 d	+4.5 d	+4.5 d	+68.2 c	+86.4 b	+90.9 b	+118 a
APX activity	0.30 d	+3.3 d	+6.7 d	+6.7 d	+70.6 c	+80.0 b	+86.7 b	+107 a
GR activity	0.34 d	+5.9 d	+2.9 d	+5.9 d	+46.7 c	+91.2 b	+88.2 b	+106 a
IAA content	12.4 b	+0.8 b	+23.4 a	+25.0 a	−41.1 e	−30.6 d	−16.1 c	+0.8 b
GA_1_ content	13.2 b	+2.3 b	+20.5 a	+20.5 a	−45.5 e	−30.3 d	−14.4 c	0 b
GA_3_ content	16.7 b	+1.2 b	+15.0 a	+16.2 a	−47.9 e	−37.7 d	−28.7 c	−1.2 b
T-Z content	11.8 b	+1.7 b	+64.4 a	+67.8 a	−42.4 e	−28.8 d	−14.4 c	+5.1 b
SOD R. Exp.	1.2 d	0 d	+8.3 d	+8.3 d	+192c	+350 b	+358 b	+467 a
CAT R. Exp.	1.2 d	0 d	0 d	0 d	+250 c	+417 b	+433 b	+533 a
APX R. Exp.	1.2 d	+8.3 d	+8.3 d	+16.7 d	+350 c	+492 b	+508 b	+600 a
GR R. Exp.	1.2 d	+16.7 d	+16.7 d	+16.7 d	+392 c	+525 b	+550 b	+667 a
PrxQ R. Exp.	1.2 d	0 d	0 d	0 d	+442 c	+600 b	+617 b	+717 a
Cd^2+^ content	ND	ND	ND	ND	+52.6 a	−43.3 b	−40.3 b	−78.3 c

Control = There is no stress and no foliar applications; Sm = foliar spray with 0.5 mM silymarin; Meg = foliar spray with 2% maize grain extract; MEg-Sm = foliar spray with maize grain extract enriched with silymarin (0.24 g Sm L^−1^ of MEg); Cd = watering the maize seedlings with nourishing solution containing 0.5 mM Cd; Cd+Sm = watering the maize seedlings with nourishing solution containing 0.5 mM Cd + foliar spray with 0.5 mM silymarin; Cd+Meg = watering the maize seedlings with nourishing solution containing 0.5 mM Cd + foliar spray with 2% maize grain extract; and Cd + MEg-Sm = watering the maize seedlings with nourishing solution containing 0.5 mM Cd + foliar spray with maize grain extract enriched with silymarin (0.24 g Sm L^−1^ of MEg). FW = fresh weight; DW = dry weight; iCE = instantaneous carboxylation efficiency; Chl. = chlorophyll; Carot. = carotenoids; O_2_^•−^ = superoxide radical; H_2_O_2_ = hydrogen peroxide; MDA = malondialdehyde; EL = ionic leakage; Pro = proline; AsA redox st. = ascorbate redox state; GSH redox st. = glutathione redox state; Sm = silymarin; SOD = superoxide dismutase; CAT = catalase; APX = ascorbate peroxidase; GR = glutathione reductase; IAA = inodole-3-acetic acid; GA_1_ = gibberellic acid 1; GA_3_ = gibberellic acid 3; T-Z = trans-zeatin; R. Exp. = relative expression; Cd = cadmium.

## Data Availability

The data presented in this study are available upon request from the corresponding author.

## Supplementary Materials

The following are available online at https://www.mdpi.com/2218-273X/11/3/465/s1, Table S1: A preliminary study conducted to assess the effect of cadmium (Cd), maize grain extract (MEg), silymarin (Sm), or silymarin-enriched maize grain extract (MEg-Sm) concentrations on some growth traits, instantaneous carboxylation efficiency (iCE), chlorophyll content, and trans-zeatin content of maize plants.
