# Supplementary material for: Silymarin-Enriched Biostimulant Foliar Application Minimizes the Toxicity of Cadmium in Maize by Suppressing Oxidative Stress and Elevating Antioxidant Gene Expression
Source: Biomolecules. 2021 Mar 21;11(3):465. doi: 10.3390/biom11030465 (PMC8004092; doi:10.3390/biom11030465)
Supplement: Supplementary file 1 [file biomolecules-11-00465-s001.pdf]

Table S1. A preliminary study conducted to assess the effect of cadmium (Cd), maize grain extract (MEg), silymarin (Sm), or silymarin-enriched maize grain extract (MEg-Sm) concentrations on some growth traits, instantaneous carboxylation efficiency (iCE), chlorophyll content, and trans-zeatin content of maize plants

Treatments	Total leaf area plant^{-1} (m^2)	Shoot dry weight plant^{-1} (g)	iCE (µmol m^{-2} s^{-1})	Chlorophyll content (mg g^{-1} FW)	trans-zeatin (ng g^{-1} FW)
Control	0.556 a	5.24 a	0.27 a	2.52 a	12.4 a
0.25 mM Cd	0.414 b	3.89 b	0.18 b	1.88 b	9.8 b
**0.50 mM Cd**	**0.294 c**	**2.39 c**	**0.11 c**	**0.89 c**	**5.9 c**
0.75 mM Cd	×××	×××	×××	×××	×××
Control	0.556 c	5.24 c	0.27 c	2.52 c	12.4 c
1% MEg	0.602 b	6.39 b	0.29 b	2.89 b	15.7 b
**2% MEg**	**0.652 a**	**7.12 a**	**0.32 a**	**3.24 a**	**19.6 a**
3% MEg	0.668 a	7.24 a	0.33 a	3.28 a	20.1 a
Control	0.556 b	5.24 c	0.27 c	2.52 c	12.4 b
0.25 mM Sm	0.590 b	6.04 b	0.29 b	2.84 b	12.9 b
**0.50 mM Sm**	**0.649 a**	**6.99 a**	**0.32 a**	**3.19 a**	**13.8 a**
0.75 mM Sm	0.654 a	7.08 a	0.32 a	3.22 a	14.1 a
Control	0.556 c	5.24 c	0.27 c	2.52 c	12.4 c
MEg-Sm (1)	0.647 b	6.84 b	0.30 b	2.91 b	14.6 b
**MEg-Sm (2)**	**0.761 a**	**8.24 a**	**0.34 a**	**3.40 a**	**18.9 a**
MEg-Sm (3)	0.772 a	8.27 a	0.35 a	3.42 a	19.0 a

The same letters with mean values±SE in each column indicate non-significant differences, and different letters indicate significant differences according to LSD test ($P \leq 0.05$). MEg-Sm (1)= 0.12 g Sm L^{-1} of MEg, MEg-Sm (1)= 0.24 g Sm L^{-1} of MEg, MEg-Sm (1)= 0.36 g Sm L^{-1} of MEg, ×××= Plants died.

This preliminary study was conducted to select appropriate concentrations of Cd, MEg, Sm, and MEg-Sm for the main study. The data in **Table S1** show that 2% MEg, 0.50 mM Sm, and MEg-Sm (2) conferred the best results economically. On the other hand, 0.75 mM Cd caused plant death, and thus, 0.5 mM Cd with biostimulant concentrations, which conferred the best economic results were selected for the main study.
